# SHP-1 acts as a tumor suppressor by interacting with EGFR and predicts the prognosis of human breast cancer

**DOI:** 10.20892/j.issn.2095-3941.2020.0501

**Published:** 2021-10-01

**Authors:** Qian Geng, Ruiting Xian, Yinjue Yu, Fengsheng Chen, Rong Li

**Affiliations:** 1 Department of Oncology, Nanfang Hospital, Southern Medical University, Guangzhou 510515, China; 2 Cancer Center, the Affiliated Changzhou No. 2 People’s Hospital of Nanjing Medical University, Changzhou 213003, China; 3 Integrated Hospital of Traditional Chinese Medicine, Southern Medical University, Guangzhou 510315, China

**Keywords:** SHP-1, EGFR, breast cancer, proliferation, migration

## Abstract

**Objective:**

The aims of this study were to examine the prognostic value of SHP-1 in breast cancer, its roles in the regulation of breast cancer cell growth and metastasis, and the underlying mechanisms.

**Methods:**

Tumor specimens from 160 patients with breast cancer and 160 noncancerous tissues were used to examine the expression of SHP-1 and to analyze its association with overall survival through Kaplan–Meier and multivariate Cox regression analyses. RNA sequencing data and the expression and clinical importance of SHP-1 in breast cancer were evaluated with data from The Cancer Genome Atlas. *In vitro* and *in vivo* assays were performed to elucidate the effects of SHP-1 on breast cancer cell proliferation and invasion. Confocal immunofluorescence and GST pulldown assays were used to demonstrate the interaction between SHP-1 and epidermal growth factor receptor, as well as its downstream pathways. Immunohistochemistry and The Cancer Genome Atlas database were used to investigate the clinical association between SHP-1 and EGFR in human breast cancer.

**Results:**

SHP-1 expression was associated with better survival in patients with breast cancer, whereas SHP-1 expression was negatively correlated with EGFR in human breast cancer. Ectopic SHP-1 expression significantly suppressed breast cancer cell proliferation, migration, and invasion. SHP-1 knockdown induced a more invasive phenotype and accelerated cell growth. Mechanistically, EGFR, a protein directly interacting with SHP-1, mediates the SHP-1-induced inactivation of Ras/Erk/GSK3β signaling and its downstream effectors.

**Conclusions:**

SHP-1 is an important prognostic biomarker in patients with breast cancer, and the SHP-1-EGFR axis is a promising target for treatment.

## Introduction

Breast cancer is the leading cause of cancer death among women 20–59 years of age, according to cancer statistics reported in 2018^1^. Given its increasing incidence, breast cancer incidence is expected to continue to be a major cause of cancer death in women worldwide^2,3^. Given the high rates of recurrence and metastasis, novel therapeutic treatments for breast cancer that are effective and safe are urgently needed.

The intricate equilibrium of phosphotyrosine residues in proteins is controlled by the opposing actions of protein tyrosine kinases (PTKs) and protein tyrosine phosphatases (PTPs)^4,5^. PTPs serve as antagonists of TK signaling that play a prominent role in cell proliferation, differentiation, invasion, and transformation^6^. Increased PTK activity or decreased PTP activity is regarded as an important cause of breast cancer development.

The epidermal growth factor receptor (EGFR) family, an essential component in PTKs activation, includes 4 members: human epidermal growth factor receptor (HER) 1 (also known as EGFR), HER2, HER3, and HER4. HER2 is overexpressed in approximately 20%–25% of breast cancers^7–9^. Overexpression of HER2 often leads to distant metastasis of breast cancers, and breast cancers often develop resistance within 1 year to the targeted drug trastuzumab^10–14^.

Much attention has been paid to therapy targeting PTKs for years, yet the importance of PTPs has been ignored. Can excessive intracellular tyrosine phosphorylation be decreased by activating PTPs and restoring the original phosphorylation balance, thus inhibiting cell proliferation, migration, and invasion?

The SHP-1 gene, also called PTPN6, HCP, SH-PTP1, or PTP1C, is located on human chromosome 12p13. It encodes a 68 kDa nonreceptor type PTP containing 2 tandem Src homology (SH2) domains, a catalytic domain, and a COOH-terminal tail of 100 amino acid residues^15^. SHP-1 is expressed in hematopoietic and epithelial cells^16–18^. In hematopoietic cells, SHP1 usually dephosphorylates several growth factor receptors, thus functioning as a negative regulator in signal transduction^19–21^. However, little is known about the functional role and prognostic importance of SHP1 in the malignant transformation process in breast cancer.

Patients who are resistant to trastuzumab can still benefit from lapatinib, a dual tyrosine kinase inhibitor (TKI) that targets both EGFR and HER2^22,23^. In addition, a phase I study of neratinib, an EGFR, HER2, and HER4 inhibitor, has shown clinical activity in breast cancer. Currently, neratinib is in phase III clinical development for the treatment of patients with breast cancer, and promising antitumor activity has been observed^24,25^. These findings together indicate that an activated bypass might exist under conditions of inhibition of HER2 by trastuzumab alone, possibly involving EGFR and HER3. Patients with breast cancer may further benefit from blocking of the entire EGFR family.

Our previous studies have shown that SHP-1 interacts with EGFR, according to coimmunoprecipitation analysis^26^. In this report, we observed that SHP-1, a tumor suppressor, directly interacts with EGFR and suppresses its induction of Ras/Erk/GSK3β signaling and its downstream cell cycle and EMT signals.

## Materials and methods

### Chemicals and reagents

3-(4,5-Dimethylthiazol-yl)-2,5-diphenyltetrazolium bromide (MTT), propidium iodide (PI) and other chemicals were purchased from Sigma (St. Louis, Missouri, USA). The specific anti-SHP1 short interfering RNA (siRNA) and nonspecific control siRNA sequences were purchased from GenePharma (Shanghai, China). Commercial antibodies to the following antigens were as follows: SHP-1, EGFR, p-GSK3β (Ser9), Cyclin D1, and c-Myc (Epitomics, Burlingame, USA); p-EGFR, β-actin, GAPDH, histone H3, and EGFR (Santa Cruz Biotechnology, California, USA); h-Ras, p-Erk1/2, Erk1/2, β-catenin, Snail, E-cadherin, and N-cadherin (Cell Signaling Technology, Massachusetts, USA); k-Ras, GSK3β (Proteintech Group, Chicago, USA); and SHP-1 (Abcam, Cambridge, UK).

### Cell lines and sample collection

The MDA-MB-231 and MCF-7 human breast cancer cell lines were cultured in RPMI 1640 medium (HyClone) containing 10% fetal bovine serum (Thermo). Both cell lines were incubated in a humidified chamber with 5% CO_2_ at 37 °C. A total of 160 paraffin-embedded breast cancer specimens and 160 adjacent normal tissue samples were obtained from the Nanfang Hospital of Southern Medical University, Guangzhou, China. The patients were 160 women between 28 and 83 years of age. Prior consent from the patients and approval for using these clinical materials for research purposes was granted by the Ethics Committees of Nanfang Hospital (Approval No. NFEC-2017-020). All specimens had a confirmed pathological diagnosis and were classified according to the American Joint Committee on Cancer (AJCC) criteria.

### Construction of the pLVX-CMV-SHP1-mCMV-GFP-PKG-puro vector and lentivirus infection

SHP1 was cloned into the pLVX-CMV-mCMV-GFP-PKG-puro lentivirus vector (Biowit Technologies, Shenzhen, China). The resulting lentivirus vector was cotransfected into 293FT cells for 84 h with Lipofectamine 2000 (Invitrogen, Carlsbad, CA, USA) to generate a lentiviral stock. MDA-MB-231 and MCF-7 cells were infected with lentiviral particles containing specific or negative control vectors, and colonies with GFP expression were selected to expand the culture for further investigation.

### Gene knockdown

RNA interference was performed by transfection of SHP-1-siRNA targeting SHP-1 (5′-GGUGGAGCAUUUCAAGAAGTT-3′) at a final concentration of 100 nM into MDA-MB-231-SHP1 and MCF-7-SHP1 cells at 30%–50% confluence with TurboFect siRNA Transfection Reagent (Fermentas, Vilnius, Lithuania) according to the manufacturer’s protocol. Cells were collected after 48–72 h for further experiments.

### Preparation of nuclear extracts

Nuclear extracts were prepared as described in the instructions of the NE-PER^TM^ Nuclear and Cytoplasmic Extraction Reagents (Thermo, Pierce Biotechnology, Massachusetts, USA). Briefly, the cell pellet was resuspended in cytosolic buffer on ice for 15 min, and after centrifugation at 16,000× *g* for 5 min, the supernatants were collected as cytoplasmic extracts. The nuclear pellets were resuspended in 50 μL of extraction buffer and incubated at 4 °C with occasional vortexing for 40 min. The mixture was finally centrifuged at 16,000 rpm for 10 min. The supernatant was collected, and its protein concentration was then measured.

### Western blot analysis

Cells were lysed in RIPA buffer [50 mM Tris-HCl, pH 8.0; 1 mM ethylenediaminetetraacetic acid, pH 8.0; 5 mM DTT; and 2% sodium dodecyl sulfate (SDS)], and the protein concentration was determined with BCA assays (Beyotime, Beijing, China). Total protein (30 μg) was resolved on 10% SDS–polyacrylamide gels and electrotransferred to polyvinylidene fluoride membranes (Invitrogen). The membranes were blocked with 3% BSA in Tris-buffered saline (pH 7.5), then immunoblotted overnight at 4 °C with the primary antibody. Horseradish peroxidase-conjugated anti-rabbit or anti-mouse IgG antibodies were used as secondary antibodies (Zhongshan, Beijing, China). Signals were detected with enhanced chemiluminescence reagents (Pierce, Rockford, IL, USA). β-actin, GAPDH, or histone H3 was used as a loading control.

### Immunohistochemistry and evaluation of staining

Paraffin sections (4 μM) from the samples were deparaffinized in 100% xylene and rehydrated in a descending ethanol series and water according to standard protocols. Heating antigen retrieval was performed in 10 mM citrate buffer for 2 min at 100 °C. Endogenous peroxidase activity and nonspecific antigens were blocked with peroxidase-blocking reagent containing 3% hydrogen peroxide and serum, and this was followed by incubation with rabbit anti-human SHP-1 (1:50, Epitomics) and anti-EGFR (1:100, Epitomics) overnight at 4 °C. After being washed, the sections were incubated with biotin-labeled goat anti-rabbit antibody for 20 min at room temperature and subsequently incubated with streptavidin-conjugated horseradish peroxidase (Maixin, Fuzhou, China). The peroxidase reaction was developed with 3,3-diaminobenzidine chromogen solution in DAB buffer substrate. Sections were visualized with DAB and counterstained with hematoxylin, mounted in neutral gum, and analyzed and under a bright field microscope. The immunohistochemistry (IHC) results were assessed by 2 independent pathologists who were blinded to the sample origin and the clinical outcome.

The staining results for the SHP-1 protein were semiquantitatively expressed as an immunohistochemical score combined with the percentage of tumor cells showing specific immunoreactivity. Each specimen was assigned a score according to the percentage of positive cells (none, 0; 1%–9%, 1; 10%–50%, 2; or 51%–100%, 3) and staining intensity (none, 0; weak, 1; moderate, 2; or strong, 3)^27,28^. The staining intensity and average percentage of positive tumor cells were assayed for 10 independent high magnification (×400) fields. The percentage positive cells and staining intensity were multiplied, and the samples were then classified into 2 groups according to their overall score: low expression, 0–4 points and high expression, 6–9 points.

### Transcriptomic analyses

Breast cancer expression profile data were obtained from The Cancer Genome Atlas (TCGA) database, and data mining and bioinformatics analysis were performed in R software with the DESeq R software package. The gene chip expression profile data were filtered and standardized to obtain the corresponding differentially expressed genes in the breast cancer and paracancerous tissues. The screening conditions for the differentially expressed genes were set to *|log2foldchange|* > 1 and *p-adjust* < 0.05.

Survival analysis was performed with the survival package in R software. Data were grouped by the median gene expression, and those greater than or equal to the median were defined as the high expression group, and those lower than the median were defined as the low expression group. The log-rank method was used to test for a difference in survival between groups, and *P* < 0.05 was considered statistically significant.

### Cell cycle analysis

For cell cycle analysis, the cells were harvested and fixed in 70% ice-cold ethanol overnight at 4 °C. The fixed cells were washed twice with phosphate buffered saline (PBS) and resuspended in 0.5 mL PBS containing 0.5 mg/mL RNase and 10 mg/mL PI at 37 °C in the dark for 30 min. The DNA content of the labeled cells was assessed with a flow cytometry assay (BD Biosciences, Franklin Lakes, NJ, USA). Each experiment was performed in triplicate.

### Apoptosis assays

Apoptosis was assessed with an Annexin V-APC/7-ADD Apoptosis Detection Kit (KeyGEN Biotech Corporation, Nanjing, China). Briefly, 1–5 × 10^5^ cells were collected, washed twice in cold PBS, and resuspended in 500 μL binding buffer. The cell suspension was stained with 5 μL Annexin V-APC and 7-ADD and then incubated for 10 min at room temperature in the dark. Apoptotic cells were assessed with FACS (BD Biosciences). Experiments were performed in several replicates to evaluate the degree of apoptosis on the basis of the extent of phosphatidylserine externalization.

### EdU incorporation assay

Cells were cultured in 96-well plates at 8 × 10^3^ cells per well overnight and then exposed to 50 μmol/L 5-ethynyl-20-deoxyuridine (EdU, RiboBio, Guangzhou, China) for an additional 2 h at 37 °C. The cells were fixed with 4% formaldehyde for 30 min and then treated with 0.5% Triton X-100 for 10 min at room temperature. After being washed 3 times in PBS, the cells from each well were exposed to 100 μL 1 × Apollo reaction cocktail for 30 min. Subsequently, the DNA from the cells in each well was stained with 100 μL of 4′, 6-diamidino-2-phenylindole (DAPI) for 10 min in the dark and visualized under a fluorescence microscope.

### Cell proliferation analysis

Cell proliferation was measured with MTT assays. Briefly, 1 × 10^3^ cells were seeded into a 96-well plate in quadruplicate and cultured for 7 days. Cells cultured in 96-well plates were treated with 20 μL MTT (5 mg/mL). After incubation at 37 °C for 4 h, the MTT medium was removed by aspiration, and 150 μL DMSO (Sigma) was added to each well. After incubation at 37 °C for an additional 10 min, the A490 values of each sample were measured with a plate reader. Experiments were performed 3 times.

### Colony formation assays

Cells were plated in triplicate at 200 cells per well in 6-well plates. Each cell group occupied 3 wells. After incubation for 12 days at 37 °C, the cells were washed twice with PBS and stained with Giemsa solution (Sigma Aldrich Corporation, St. Louis, MO, USA). The number of colonies containing ≥ 50 cells was counted under a microscope. The colony formation efficiency was calculated as: (number of colonies/number of cells inoculated) × 100%.

### Wound healing assays

Cells (5 × 10^5^) were cultured in 6-well tissue culture plates to confluence. A pipette tip (100 μL) was used to scratch a wound in the middle of the culture well, and the cells were cultured in RPMI 1640 supplemented with 3% serum at 37 °C. Cell migration was evaluated on the basis of the differences in the widths of the wounds at 0 h, 24 h, and 48 h.

### Cell migration and invasion assays

For *in vitro* cell migration assays, 1 × 10^5^ cells in 100 μL of the corresponding medium without fetal calf serum (FCS) were seeded on a fibronectin coated polycarbonate membrane insert in a Transwell apparatus (Costar, Massachusetts, USA). In the lower chamber, 500 μL complete growth medium was added as a chemoattractant. After 8 or 12 h of incubation, the insert was washed with PBS, and the cells on the top surface of the insert were removed with a cotton swab. Cells adhering to the lower surface were fixed with methanol, stained with Giemsa solution, and counted under a microscope in 5 predetermined fields (100×). All assays were independently repeated at least 3 times. For the cell invasion assays, the procedure was similar to those for the cell migration assay, except that the Transwell membranes were precoated with 24 μg/μL Matrigel (R&D Systems, Minnesota, USA). Cells adhering to the lower surface were counted in the same manner as in the cell migration assay.

### Immunofluorescence staining of cells

Cells were grown on cell culture dishes overnight in a 37 °C culture incubator. Before immunofluorescence staining, the cells were fixed in 4% formaldehyde for 10 min and treated with 0.5% Triton X-100 for 5 min at room temperature. The cells were then incubated with primary antibodies against EGFR (1:50, Santa Cruz Biotechnology) and SHP-1 (1:20, Abcam) in PBS with 1.5% normal serum at 4 °C overnight. After the cells were washed twice with PBS, fluorophore-conjugated secondary antibodies were diluted in PBS and incubated with the cells in the dark at room temperature for 45–60 min. Finally, the cells were incubated for 10 min with DAPI. Fluorescently stained cells were examined under a laser confocal microscope.

### *In vivo* proliferation assay

All *in vivo* experiments were conducted in accordance with the principles and procedures outlined in the Southern Medical University Guide for the Care and Use of Animals under assurance number SCXK (Guangdong) 2008-0002. The research was reviewed and approved by the institutional ethical committee before the study was conducted. The approval number was NFYY-2014-03. To determine the effects of SHP-1 on breast cancer cell line proliferation *in vivo*, we injected 1 × 10^6^ cells of MDA-MB-231 and 1 × 10^7^ cells of MCF-7 subcutaneously into 3- to 4-week-old nude mice (MCF-7, *n* = 8; MDA-MB-231, *n* = 5; Medical Laboratory Animal Center, Guangdong, China), and studied tumor progression over time. Six days after the cell injection, we began to measure the tumor sizes. The tumor length and width were measured with calipers every 3 days, and the tumor volume was calculated with the formula V = 1/2(width^2^ × length). After 18 days, the mice were sacrificed, and the tumor tissues were excised and weighed.

### Construction, expression, and purification of fusion protein

The truncated SHP-1 cDNA was amplified by PCR from pGEX-SHP1 with the appropriate primers flanked by appropriate restriction sites and ligated in frame into the pGEX vector. GST and GST-SHP1 fusion proteins were expressed in *E. coli* BL21 cells and induced with 1.0 mM isopropyl-beta-D-thiogalactopyranoside (Promega) for 16 h at 20 °C. Bacteria were resuspended in lysis buffer B (50 mM Tris, pH 7.4, 150 mM NaCl, 1% NP-40, 0.5% sodium deoxycholate, 0.1% SDS, 1 mM EDTA, and 1 mM PMSF) containing 100 μg/mL lysozyme. The bacterial extracts were sonicated for 20 min and centrifuged at 12,000 rpm for 30 min at 4 °C to remove cell debris. GST and GST-SHP1 fusion proteins were purified from the bacterial lysates by affinity chromatography with glutathione Sepharose 4B (GE Healthcare). The purified protein concentrations were estimated with BCA assays. Then equal amounts of these fusion proteins were used for subsequent GST pull-down assays.

### GST pull-down assays

Whole cell extracts (0.5 mg of protein) were incubated overnight with glutathione Sepharose beads coated with GST or GST-SHP1 (50 μg of recombinant protein) at 4 °C on a rocker. Specific antibodies against EGFR were used for detection. The levels of recombinant GST and GST-SHP1 were analyzed with Coomassie Brilliant Blue staining of the SDS-PAGE gels.

### Statistical analysis

All quantified data represented an average of at least 3 samples. SPSS 16.0 and GraphPad Prism 5.0 software were used for statistical analysis. All data are expressed as the mean ± SD. Significance was established with two-tailed Student’s t-test or one-way ANOVA as appropriate. Significant associations between SHP-1 expression and clinicopathologic parameters were assessed with the chi-square test. Kaplan–Meier and log-rank tests were used to compare patient survival and to create survival curves based on the SHP-1 or EGFR IHC scores. Multivariate survival analysis was performed for all parameters that were significant in the univariate analysis with the Cox regression model. *P* < 0.05 was considered statistically significant.

## Results

### SHP-1 is associated with better survival in human breast cancer

We measured the expression of SHP-1 protein in 160 archived paraffin-embedded breast cancer samples and 160 adjacent noncancerous specimens through immunohistochemical staining. SHP-1 was highly expressed in 74.4% (119/160) of adjacent normal breast tissues but only 56.25% (90/160) of breast cancer tissues (*P* < 0.0001; **Supplementary Table S1**).

**Figure 1:**
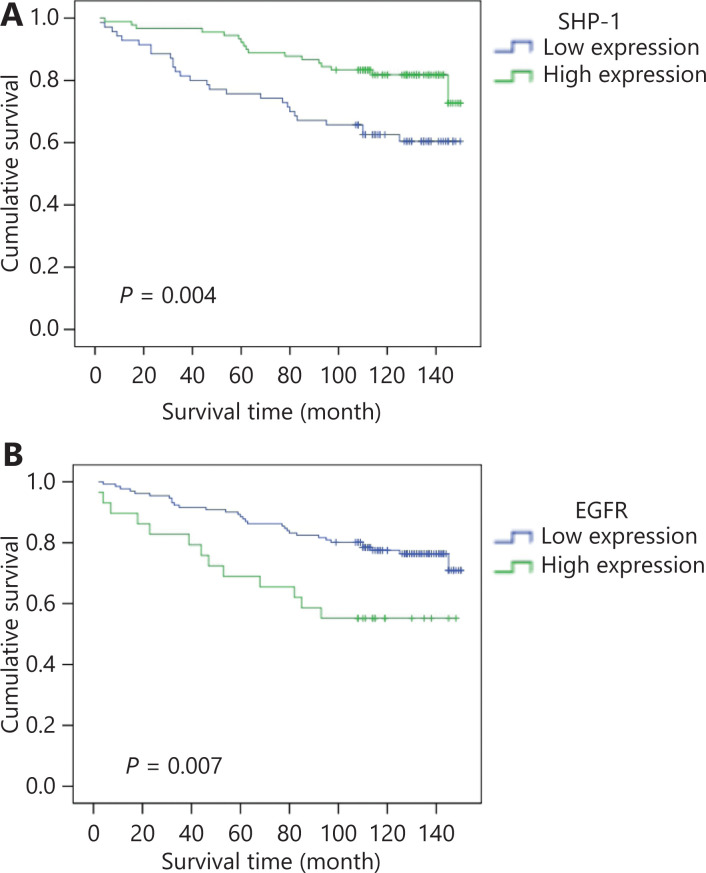
Kaplan–Meier analysis of the expression of SHP-1 and EGFR. (A) Patients with higher expression of SHP-1 had longer overall survival (OS, *P* = 0.004). (B) Patients with lower expression of SHP-1 had longer overall survival (OS, *P* = 0.007). EGFR was inversely associated with patient survival and thus predicted poor prognosis.

Furthermore, we investigated the prognostic value of SHP-1 expression in 160 patients with breast cancer with 150 months of follow-up information. Kaplan–Meier analysis showed that the patients with higher levels of SHP-1 had longer overall survival (OS, *P* = 0.004; **Table 1** and **Figure 1A**). Notably, the patients with breast cancer could be divided into 2 subgroups with different outcomes according to SHP-1 expression, and higher SHP-1 expression was associated with a better patient prognosis OS (*P* = 0.004).

**Table 1:** Univariate prognostic factors in Kaplan–Meier survival analysis (*n* = 160)

Characteristics	*n*	Deaths	Mean	SE	Log-rank	*P*
Age (years)						
≤50	76	16	128.630	5.094	2.687	NS
>50	84	28	119.696	5.080		
Tumor size (cm)						
T1 (≤2)	36	9	126.889	6.230	2.477	NS
T2 (2–5)	109	29	126.098	4.106		
T3 (>5)	15	6	94.600	16.976		
TNM stage						
I	15	3	138.317	4.919	7.773	0.021*
II	96	21	130.343	3.965		
III	49	20	105.656	8.066		
Histological grade						
G1–G2	156	40	125.419	3.596	14.014	<0.001**
G3	4	4	70.250	26.110		
Lymph node invasion						
N0	66	16	130.921	4.441	8.680	0.034*
N1	49	9	129.939	5.693		
N2	37	16	104.480	9.084		
N3	8	3	102.750	20.786		
ER						
Negative	58	23	109.566	6.746	7.721	0.005**
Positive	102	21	131.215	3.947		
PR						
Negative	81	28	115.971	5.649	4.519	0.034*
Positive	79	16	132.083	4.302		
HER2						
Negative	117	34	123.780	4.144	0.307	NS
Positive	43	10	124.488	7.355		
SHP-1						
Low expression	70	27	111.209	6.364	8.253	0.004**
High expression	90	17	133.801	3.795		
EGFR						
Low expression	131	31	128.792	3.619	7.284	0.007**
High expression	29	13	101.138	10.370		
Group						
SHP-1 high, EGFR low	81	13	135.984	3.834	13.072	0.004**
SHP-1 low, EGFR low	50	18	117.094	6.852		
SHP-1 high, EGFR high	9	4	112.778	13.909		
SHP-1 low, EGFR high	20	9	94.250	13.192		

*P < 0.05; **P < 0.01.

Univariate analysis showed that TNM stage, histological grade, lymph node metastasis, ER status, and PR status were also significantly correlated with patient survival (*P* = 0.021, *P* < 0.001, *P* = 0.034, *P* = 0.005, and *P* = 0.034, respectively; **Table 1**). To determine whether SHP-1 was an independent prognostic factor for breast cancer, we performed multivariate analysis of SHP-1 protein expression levels adjusted for TNM stage, histological grade, lymph node metastasis, ER status, and PR status, by using a Cox proportional hazard model. Decreased SHP-1 expression was found to be a weak independent prognostic factor for OS in patients with breast cancer (*P* = 0.036; **Supplementary Table S2**).

We also determined the relationship between SHP-1 expression and the clinicopathological characteristics of 160 breast cancer tissue samples with available associated clinical details (**Supplementary Table S3**). There were no significant associations between SHP-1 expression and patient age, histological grade, or TNM stage. However, SHP-1 expression was inversely correlated with the expression of EGFR (*P* = 0.003), but positively correlated with the expression of ER (*P* = 0.013) and PR (*P* = 0.04). These data indicated an inverse association of SHP-1 expression with breast cancer malignancy and revealed that SHP-1 may be a potential biomarker for prognosis in patients with breast cancer.

### SHP-1 inhibits the proliferation of breast cancer cells *in vitro* and *in vivo*

The above observations prompted us to explore the potential biological function of SHP-1 in breast cancer progression. First, we determined that the basal expression level of SHP-1 protein in the 2 cell lines was moderate in MCF-7 and almost absent in MDA-MB-231. Second, we introduced SHP-1 into MDA-MB-231 and MCF-7 cells by using lentivirus to upregulate SHP-1 expression. The SHP-1 protein levels were observed by Western blot (**Supplementary Figure S1A**). Then we detected the effect of siRNA knockdown of SHP-1 expression, as compared with the results in the negative control (MCF-7-SHP-1-siNC and MDA-MB-231-SHP-1-siNC) groups (**Supplementary Figure S1A**).

**Figure 2:**
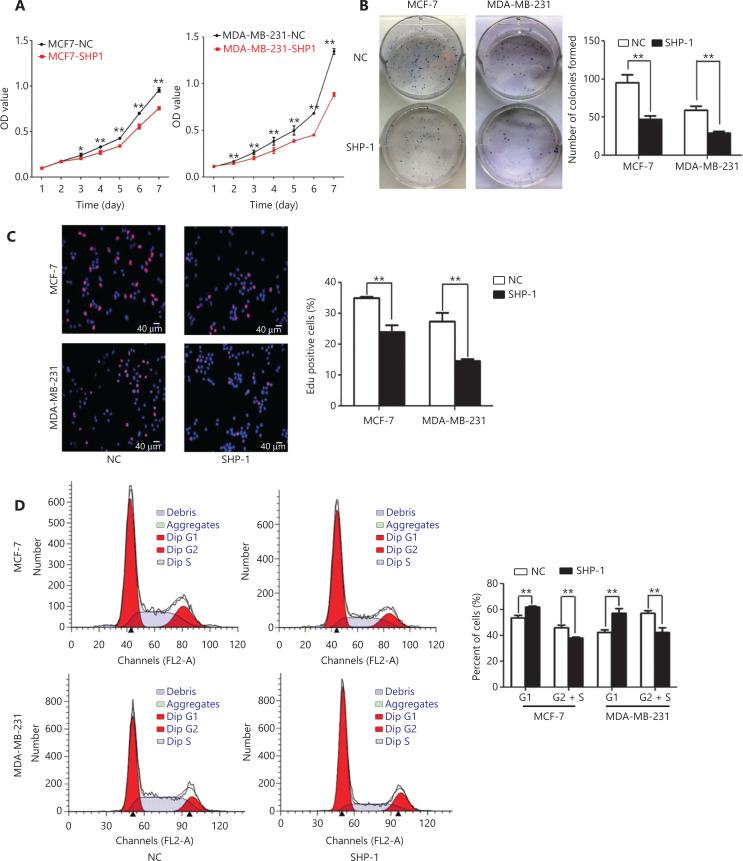

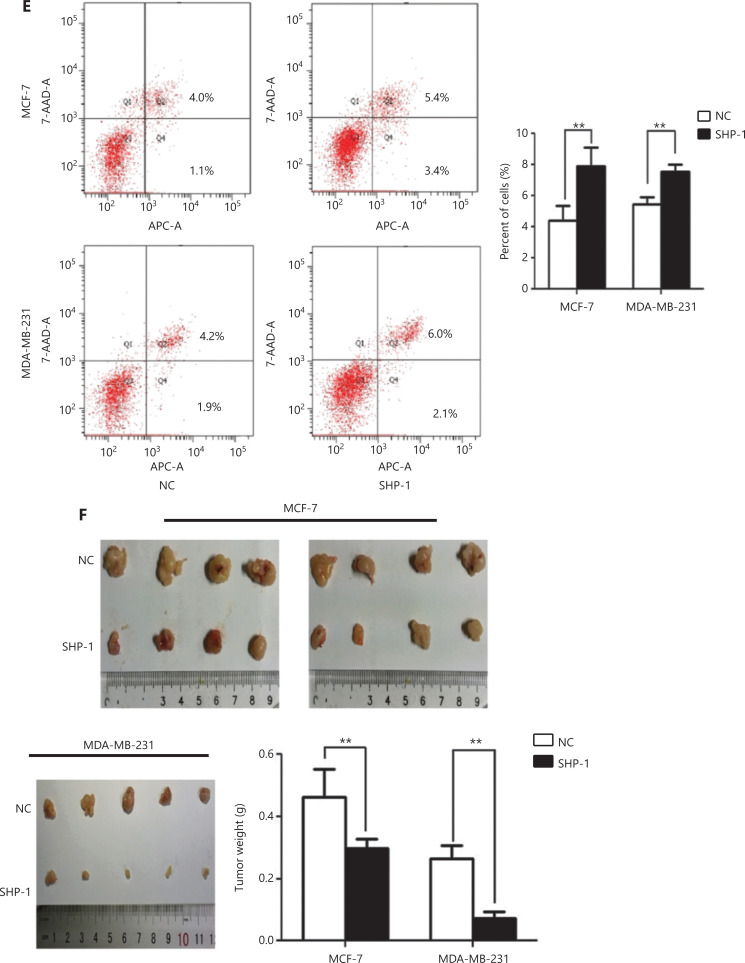
SHP-1 inhibited the proliferation of breast cancer cells *in vitro* and *in vivo*. (A) MTT assays on MCF-7-SHP1 and MDA-MB-231-SHP1 cells. SHP-1 overexpression inhibited proliferation in stable MCF-7 and MDA-MB-231 cell lines compared with control cells. Data are presented as mean ± SD for 3 independent experiments. (B) Colony formation assays on MCF-7-SHP1 and MDA-MB-231-SHP1 cells. SHP-1 upregulation significantly decreased the number of MCF-7 and MDA-MB-231 colonies in culture, as compared with controls. Data are presented as mean ± SD for 3 independent experiments. (C) EdU incorporation assays on MCF-7-SHP1 and MDA-MB-231-SHP1 cells revealed that the percentage of cells in S phase decreased after upregulation of SHP-1. (D) MCF-7 and MDA-MB-231 cells were fixed and then stained with PI for flow cytometry analysis. The phase percentages of G1, and G2 + S are displayed in a bar graph. The data represent mean ± SEM (*n* = 3). (E) The overexpression of SHP-1 promoted apoptosis in MDA-MB-231-SHP1 and MCF-7-SHP1 cells compared with control cells. (F) The results of an *in vivo* tumorigenesis study in nude mice. Experiments were performed on 8 mice for the MCF-7 group and 5 mice for the MDA-MB-231 group. Weights of the xenografts excised from the tumor bearing mice at day 18. Compared with negative control cells, SHP1-overexpressing cells showed markedly less tumorigenicity *in vivo*. *P < 0.05; **P < 0.01 by Student *t* test.

Subsequently, we evaluated the effects of SHP-1 on cellular proliferation through MTT, colony formation, and EdU incorporation assays *in vitro*. SHP-1 overexpression inhibited the proliferation of the MCF-7 and MDA-MB-231 cells, whereas SHP-1 knockdown promoted the proliferation of transiently transfected MCF-7-SHP1 and MDA-MB-231-SHP1 cells (**Figure 2A and Supplementary Figure S1B**). Colony formation assays showed that SHP-1 upregulation resulted in significantly fewer MCF-7 and MDA-MB-231 colonies than control colonies (**Figure 2B**). EdU incorporation assays revealed that the percentage of cells in S phase decreased after upregulation of SHP-1 but increased after downregulation of SHP-1 (**Figure 2C and Supplementary Figure S1C**). In addition, we quantified the cell cycle distribution with flow cytometry and found that the number of cells slightly increased at G1 phase and significantly decreased at S phase in SHP-1-overexpressing MCF-7 and MDA-MB-231 cells (**Figure 2D**). Flow cytometry analysis with Annexin V-APC/7-AAD staining demonstrated that cell apoptosis was induced by up-regulation of SHP-1 in MDA-MB-231 and MCF-7 cells compared with control cells (**Figure 2E**).

To further confirm the growth-suppressive effect of SHP-1, we performed an *in vivo* tumorigenesis study in nude mice. The average volume and weight of the subcutaneous tumors resulting from MCF-7-SHP1 and MDA-MB-231-SHP1 cell injection were significantly lower than those resulting from MCF-7-NC and MDA-MB-231-NC cell injection (**Figure 2F and Supplementary Figure S1D**). IHC examination indicated greater SHP-1 expression in MCF-7-SHP1 and MDA-MB-231-SHP1 xenograft tumor specimens compared with negative control specimens (**Supplementary Figure S1E**). These results suggested a significant *in vivo* inhibitory effect of increased SHP-1 on tumorigenesis.

### SHP-1 suppresses breast cancer cell migration and invasion *in vitro*

**Figure 3:**
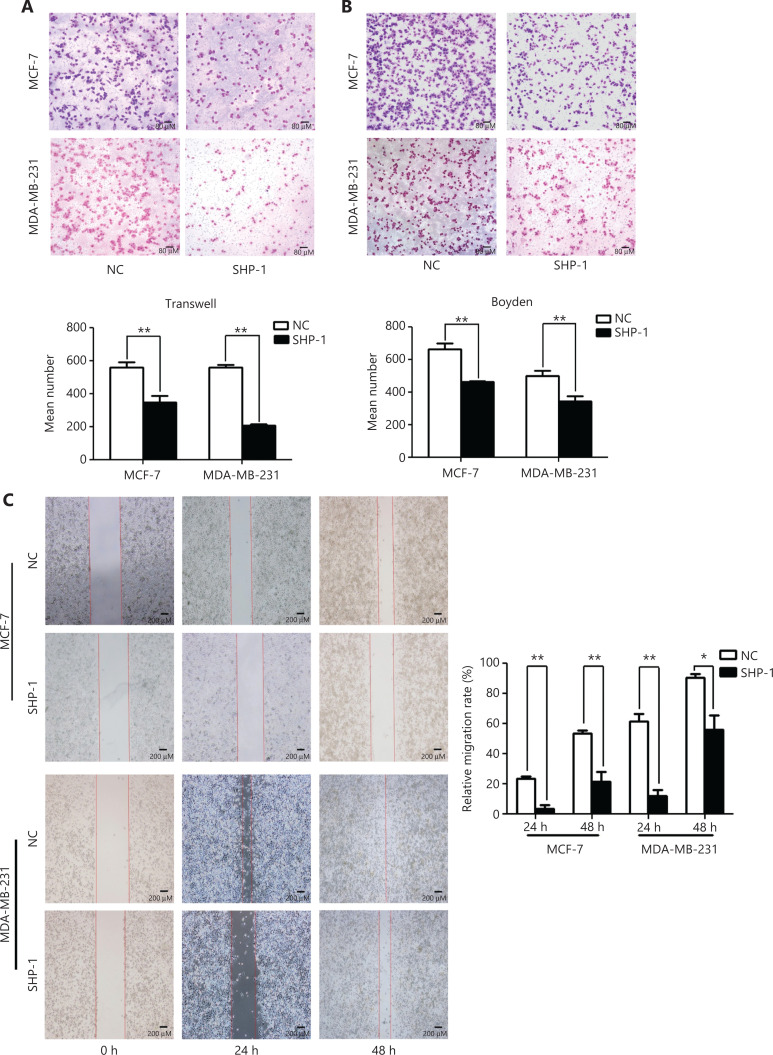
SHP-1 suppressed breast cancer cell migration and invasion *in vitro*. (A) Transwell chamber assays. The percentage of cells that migrated in both the MDA-MB-231-SHP1 and MCF-7-SHP1 cell groups was dramatically lower than that in the control group. (B) Boyden chamber assays. Increased SHP-1 expression significantly decreased the invasion activity of MDA-MB-231 and MCF-7 cells on Matrigel. (C) Wound-healing assays showed the impaired migration associated with SHP-1 overexpression. Data are presented as mean ± SD for 3 independent experiments. *P < 0.05; **P < 0.01 by Student *t* test.

To determine the function of SHP-1 in breast cancer cell migration and invasion, we performed Transwell, wound-healing, and Boyden chamber assays. After incubation for 8 h and 12 h, the percentage of cells that migrated in both the MDA-MB-231-SHP1 and MCF-7-SHP1 cell groups was dramatically lower than that in the control cell group (**Figure 3A**). In addition, increased SHP-1 expression led to a significant decrease in the invasive activity of MDA-MB-231 and MCF-7 cells on Matrigel (**Figure 3B**). As evidenced by wound-healing assays (**Figure 3C**), SHP-1 overexpression impaired migration. In contrast, migration and invasion were enhanced after siRNA knockdown of SHP-1 expression in MDA-MB-231-SHP1 and MCF-7-SHP1 cells (**Supplementary Figure S2A and S2B**).

### SHP-1 specifically and directly interacts with EGFR

**Figure 4:**
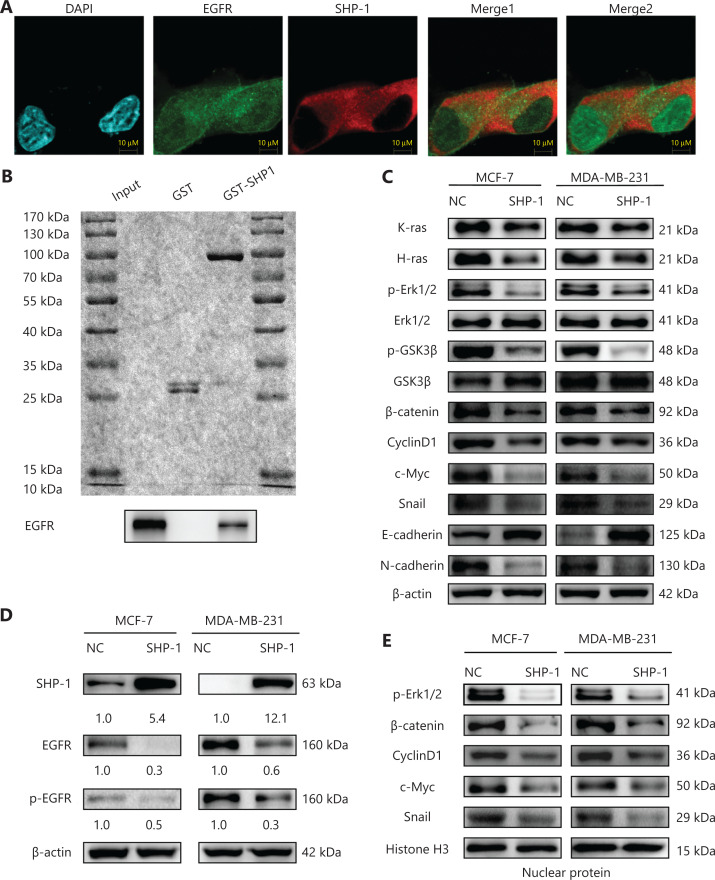
SHP-1 colocalized, interacted with, and dephosphorylated EGFR, thus inactivating the downstream Ras/Erk/GSK3β signaling pathway. (A) Confocal immunofluorescence microscopy analysis. MDA-MB-231 cells expressing endogenous EGFR were transfected with pLVX-CMV-SHP1. Cells were fixed and immunostained for EGFR (green) and SHP-1 (red), and the cell nuclei were labeled with DAPI (blue). Representative images from one of 3 separate experiments are shown. (B) GST-fusion proteins were purified, coupled to glutathione beads, and incubated with cell lysates from MDA-MB-231 cells, as indicated. Beads were then washed, and EGFR content was assessed with immunoblot analysis by using the cell lysate as input control (bottom). Equal quantities of each fusion protein were separated by 10% SDS-PAGE followed by Coomassie blue staining (top). (C, D) Western blot showed that SHP-1 overexpression resulted in downregulation of k-ras, h-ras, p-Erk1/2, p-GSK3β, β-catenin, Cyclin D1, c-Myc, Snail, and N-cadherin, and upregulation of GSK3β and E-cadherin, possibly through decreasing EGFR phosphorylation (p-EGFR). (E) The levels of p-Erk1/2, β-catenin, cyclin D1, c-Myc, and Snail were significantly lower in the SHP-1-overexpressing cells than control cells. All experiments were performed at least in triplicate.

Given our previous findings, we attempted to confirm a direct interaction between SHP-1 and EGFR. Therefore, we performed confocal immunofluorescence and GST pull-down assays. We used MDA-MB-231-SHP1 cells to analyze the subcellular distribution of SHP-1 and EGFR with confocal immunofluorescence microscopy analysis. EGFR was located throughout the whole cell, particularly in the region around the nucleus, and SHP-1 was mainly detected in the cytoplasm. The two proteins clearly colocalized in the cytoplasm, as visualized by an orange color (**Figure 4A**).

A direct interaction between SHP-1 and EGFR was confirmed with direct *in vitro* binding assays. For this purpose, GST-fusion proteins alone or in combination with full-length SHP-1 were immobilized on glutathione affinity beads and incubated with protein extracts from MDA-MB-231 cells that overexpressed EGFR. As shown in **Figure 4B**, EGFR was pulled down by SHP-1. Together, these results revealed that SHP-1 specifically and directly binds EGFR.

### SHP-1 dephosphorylates EGFR and inactivates the Ras/Erk/GSK3β signaling pathway

To further determine the mechanisms through which SHP-1 suppresses breast cancer, we focused on EGFR and members of its major downstream signaling pathway to investigate whether they might be responsible for the suppressed proliferation and invasion induced by SHP-1. As shown in **Figure 4C and 4D**, SHP-1 overexpression resulted in downregulation of k-ras, h-ras, p-Erk1/2, p-GSK3β, β-catenin, Cyclin D1, c-Myc, Snail, and N-cadherin, and upregulation of GSK3β and E-cadherin, possibly through a decrease in EGFR phosphorylation. In addition, the nuclear protein levels of p-Erk1/2, β-catenin, cyclin D1, c-Myc, and Snail were significantly lower in SHP-1-overexpressing cells than control cells (**Figure 4E**). Similar results were confirmed in subcutaneous tumor tissues of nude mice injected with breast cancer cells (**Supplementary Figure S3A**). Consistently, immunohistochemical staining of specimens from *in vivo* tumor nodules demonstrated that the protein levels of p-ERK1/2, β-catenin, cyclin D1, c-Myc, and N-cadherin were diminished, and the protein level of E-cadherin was increased by SHP-1 (**Supplementary Figure S3B**).

**Figure 5:**
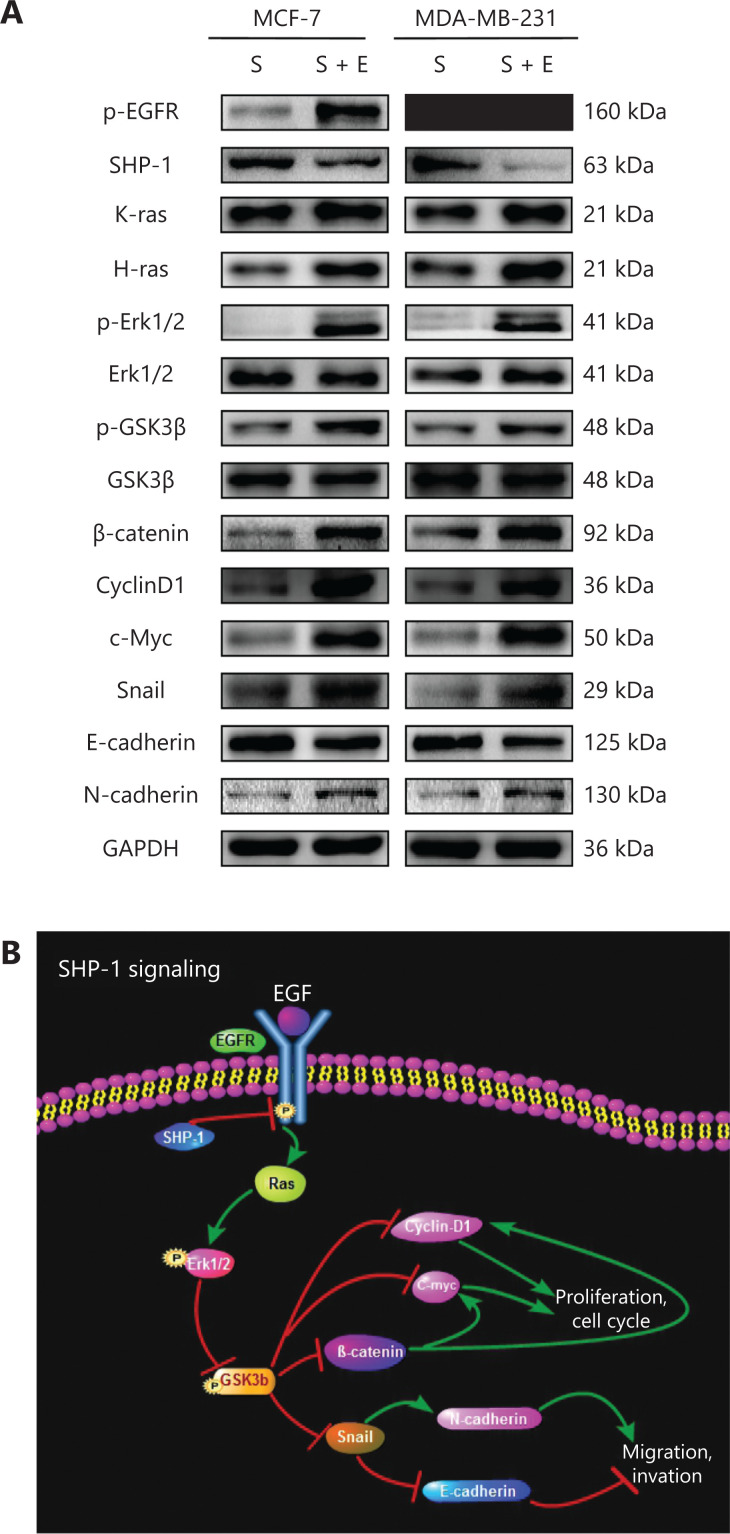
EGF stimulation reversed the efficiency of pLVX-CMV-SHP1 and the underlying mechanism involved. (A) Overexpression of SHP-1 inactivated EGFR signaling pathway in MCF-7 and MDA-MB-231 cells, and EGF stimulation reversed the efficiency of pLVX-CMV-SHP1. S, cells infected with SHP1-targeted lentiviral particles; S + E, cells infected with SHP1-targeted lentiviral particles followed by 100 ng/mL EGF treatment. Data are presented as mean ± SD for 3 independent experiments. (B) A schematic diagram showing the signaling pathway involved.

Next, we detected the relevant proteins responding to EGF stimulation in MCF-7-SHP1 and MDA-MB-231-SHP1 cells. As shown in **Figure 5A**, treatment with EGF (100 ng/mL, 6 h) significantly increased p-EGFR, h-ras, k-ras, p-Erk1/2, p-GSK3β, β-catenin, Cyclin D1, c-Myc, Snail, and N-cadherin, and decreased E-cadherin and SHP-1 protein levels, thus indicating that EGF might reverse the changes induced by SHP-1.

These results revealed that EGFR interacts with SHP-1 and inhibits activation of the Ras/Erk/GSK3β pathway, thus decreasing breast cancer cell proliferation and invasion, and consequently inhibiting breast cancer progression. We then constructed a schematic diagram to clearly demonstrate the signaling pathway revealed by the results (**Figure 5B**).

### SHP-1 is negatively correlated with EGFR in human breast cancer

**Figure 6:**
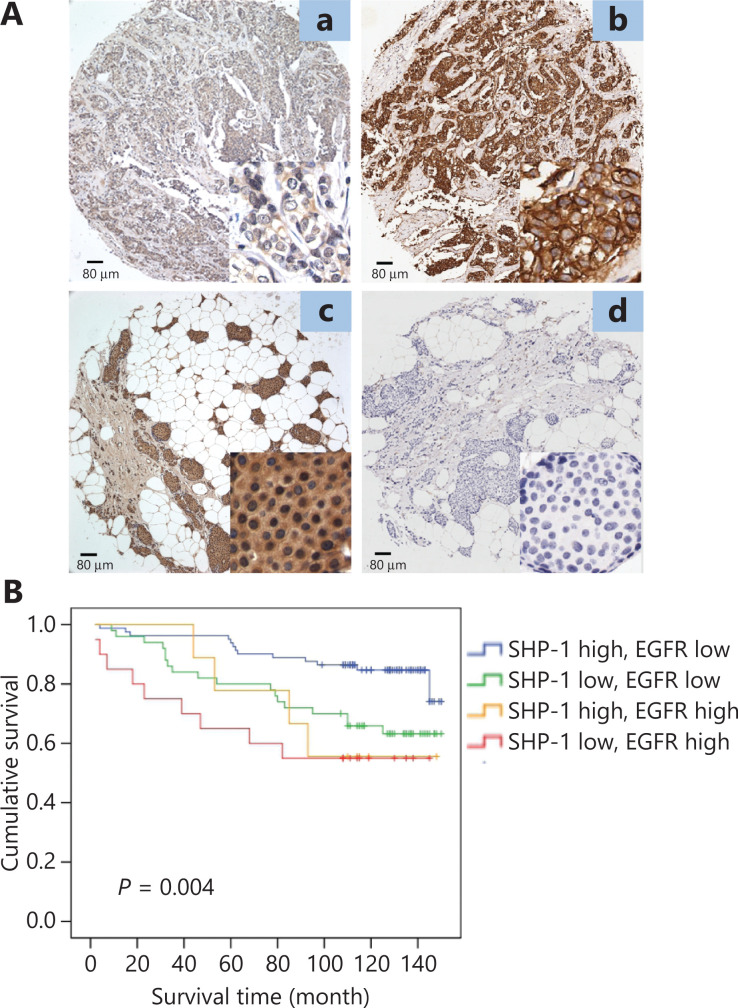
SHP-1 was negatively correlated with EGFR in human breast cancer. (A) Representative immunohistochemical images showing the expression of SHP-1 (left) and EGFR (right) in the same breast cancer tissue (a and b, c and d). a, low expression of SHP-1; b, high expression of EGFR (cell membrane); c, high expression of SHP-1 (cytoplasm); and d, low expression of EGFR. (B) Kaplan-Meier survival analysis of overall survival in 160 breast cancer patients, on the basis of SHP-1 and EGFR protein expression. High SHP-1 and low EGFR expression indicated favorable breast cancer prognosis (*P* = 0.004). The *log-rank* test was used to calculate *P* values.

The above observations prompted us to detect the expression of EGFR in patients with breast cancer. EGFR was highly expressed in 18.1% (29/160) of 160 breast cancer tissue samples. Interestingly, the expression of SHP-1 was negatively correlated with EGFR (*P* = 0.003; **Supplementary Table S3**), corresponding to the previous results observed in basic science experiments. Representative results from the IHC analysis are shown in **Figure 6A**. In addition, EGFR was inversely associated with patient survival and thus predicted a poor outcome (*P* = 0.007; **Table 1** and **Figure 1B**).

We subsequently divided all 160 breast cancer cases into 4 groups: a high SHP-1 and high EGFR expression group, a high SHP-1 and low EGFR expression group, a low SHP-1 and high EGFR expression group, and a low SHP-1 and low EGFR expression group. As shown in **Figure 6B**, the high SHP-1 and low EGFR expression group had the best survival, and the low SHP-1 and high EGFR expression group had the worst survival (*P* = 0.004).

### TCGA data reveal an association between SHP-1 mRNA expression/SHP-1 and EGFR coexpression and breast cancer prognosis

**Figure 7:**
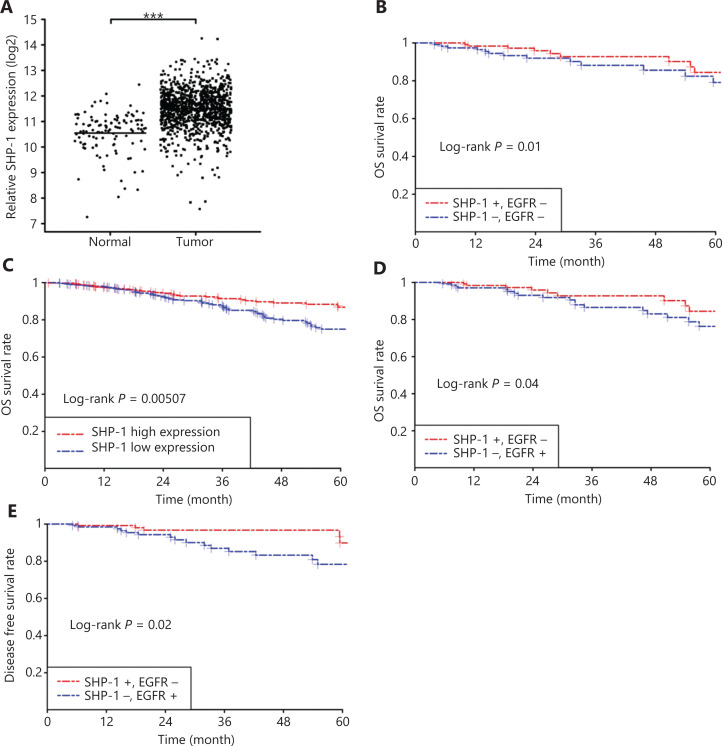
TCGA data revealed an association between SHP-1 mRNA expression/SHP-1 and EGFR co-expression levels and breast cancer prognosis. (A) Specimens from patients with breast cancer showed clearly lower SHP-1 expression than that in the matched normal specimens (*P* < 0.001). (B, C) After adjustment for known risk factors, SHP-1 was demonstrated to markedly correlate with longer overall survival (OS, *P* = 0.01). Compared with low expression, high expression of SHP-1 was closely associated with overall survival (OS, *P* = 0.00507). (D, E) High SHP-1 and low EGFR expression groups showed longer OS (*P* = 0.04) and DFS (*P* = 0.02) than that in the low SHP-1 and high EGFR expression group. ***P < 0.001.

To verify that SHP-1 was associated with better survival in human breast cancer, we first analyzed SHP-1 mRNA expression between normal tissues (*n* = 113) and cancer tissues (*n* = 1,109) according to the transcriptomic analyses available in the TCGA database. Patients with breast cancer showed clearly lower SHP-1 expression than their matched normal specimens (**Figure 7A**, *P* < 0.001). Subsequently, after adjusting for known risk factors, we demonstrated that SHP-1 was markedly correlated with an extension in overall survival (OS, **Figure 7B**, *P* = 0.01). Compared with low expression, high SHP-1 expression was closely associated with overall survival (OS, **Figure 7C**, *P* = 0.00507).

In addition, our analysis of the association between the SHP-1 and EGFR expression patterns and their prognostic value in the TCGA database was consistent with preliminary experimental results. The high SHP-1 and low EGFR expression groups showed longer OS (**Figure 7D**, *P* = 0.04) and DFS (**Figure 7E**, *P* = 0.02) than the low SHP-1 and high EGFR expression groups.

## Discussion

SHP-1, which is expressed in hematopoietic and epithelial cells, is a PTP that antagonizes the growth-promoting and oncogenic potential of tyrosine kinases^29^. SHP-1 has been proposed as a candidate tumor suppressor gene in prostate cancer, hepatocellular carcinoma, and other cancers^29–32^. The clinical relevance of SHP-1 in breast cancer remains unclear. In the present study, we identified SHP-1 as a tumor suppressor that inhibits breast cancer cell proliferation and invasion. Then we identified EGFR as a specific and direct target of SHP-1, thus improving understanding of the mechanisms underlying breast cancer progression. Moreover, we found that SHP-1 and EGFR not only were correlated with each other but also predicted the OS of patients with breast cancer, thus highlighting the value of SHP-1 as a novel prognostic biomarker in human breast cancer.

PTPs have received substantial attention since the discovery of PTPN12, which was described as a tumor suppressor in triple-negative breast cancer^33^. Analyses of PTPs have provided new perspectives for breast cancer progression, highlighting the potential value of PTPs in predicting the outcomes of patients. Tumor size, a factor incorporated in clinical staging, is one of the most important prognostic indicators for breast cancer^34^. Patients with tumors lacking expression of ER and PR tend to have poorer prognosis, with earlier and more frequent recurrence^35^. Tumor cells positive for EGFR have a high degree of malignancy and are prone to invasion and metastasis. Our present data showed that the expression of SHP-1 was significantly positively correlated with ER and PR expression levels and negatively associated with EGFR expression levels, thereby suggesting that SHP-1 plays an important role in breast cancer. Our findings also indicated that SHP-1 might dephosphorylate EGFR and consequently be associated with ER status. In addition, patients with relatively higher levels of SHP-1 had a better prognosis, thus suggesting that SHP-1 is an independent prognostic factor for breast cancer. Furthermore, SHP-1 protein can be easily detected by IHC staining, as shown in our study. The TCGA dataset showed that patients with breast tumors with high SHP-1 mRNA expression levels had longer OS and DFS than those with low expression levels. Therefore, we concluded that SHP-1 may be a novel and clinically feasible candidate for breast cancer treatment.

EGFR, a key molecule in the EGF receptor family that contains extracellular, transmembrane, and tyrosine kinase domains, plays important roles in cell proliferation, cell adhesion, cell motility, and differentiation^36–39^. We identified EGFR instead of HER2 as a protein that specifically and directly interacts with SHP-1, on the basis of coimmunoprecipitation analysis^26^, confocal immunofluorescence, and GST pull-down assays. These interesting findings place SHP-1 in a position influencing EGFR signaling and potentially altering the signaling output of EGFR. We further demonstrated that the SHP-1–induced suppression of EGFR inactivated the Ras/Erk/GSK3β pathway, thus enhancing understanding of the molecular mechanism of breast cancer progression, given that this pathway is known to be dysregulated in many cancers^40–42^. Moreover, EGF, one of the most potent mitogens that transmits signals for cell growth, survival, and motility by binding and activating EGFR^43–45^, induced an increase in h-ras, k-ras, p-Erk1/2, p-GSK3β, β-catenin, Snail, c-Myc, cyclin D1, and N-cadherin, and a decrease in E-cadherin in SHP-1-overexpressing breast cancer cells, thereby reversing the changes caused by SHP-1.

Interestingly, the effect of SHP-1 appears to be confined to the EGFR/Ras/Erk pathway without affecting the important PI3K/Akt pathway (data not shown). This observation led us to suggest that although SHP-1 might be an important factor in breast cancer, it might also be a member of an even larger constellation of elements that together drive recalcitrant malignancy. In addition, SHP-1 was found to inactivate signal transducers and activators of transcription 3 (STAT3) in HER2-overexpressing breast cancer cells (data not shown), thus indicating that the tumor-suppressive effects of SHP-1 cannot be fully compromised by EGFR/Ras/Erk/GSK3β pathway inactivation.

EGFR, which is overexpressed in approximately 30% of human primary tumors, is correlated with nodal status, tumor size, histological grade, Ki-67 index, ER/PR status, and prognosis in breast cancer, and has been a target of anti-cancer drugs^22,46–52^. Previous results have indicated a potential strong association between the expression of SHP-1 and EGFR in human breast cancer. SHP-1 was negatively associated with EGFR in both human breast cell lines and tumor specimens. In agreement with findings from previous studies, higher expression of EGFR predicted poorer clinical outcomes in our study. We then divided all 160 patients with follow-up information into 4 groups according to protein levels of SHP-1 and EGFR. As expected, the low SHP-1 and high EGFR expression groups had the poorest survival, and the corresponding high SHP-1 and low EGFR expression groups had the best survival. In addition, the TCGA data further confirmed that the high SHP-1 and low EGFR expression groups showed longer OS and DFS than the low SHP-1 and high EGFR expression groups. These results may aid in understanding of the clinical prognostic value of the coexpression patterns of SHP-1 and EGFR. They additionally underscore the need to pay more attention to patients with low SHP-1 and high EGFR expression and to apply early and aggressive interventions, because this group might have poorer prognosis.

We identified SHP-1 as a tumor suppressor that inhibits the proliferation and invasion of breast cancer cells, thereby suppressing the growth and metastasis of breast cancers and highlighting the therapeutic potential of SHP-1 in breast cancer treatment. Notably, although much work remains to be done to develop clinical applications for SHP-1 agonists, TKIs have been widely used in the treatment of many malignant tumors. Previous results have indicated that loss of SHP-1 might serve as a biomarker for the application of TKIs in patients with breast cancer, and these patients might achieve additional benefit from therapy targeted against EGFR. Together, our findings suggest that patients with lower levels of SHP-1 might benefit from agents that inhibit EGFR and that SHP-1 may be considered a biomarker for the early use of EGFR inhibitors. Further study perspectives are warranted.

## Conclusions

In summary, our current results revealed that SHP-1 significantly inhibits breast cancer cell proliferation and cell invasion and suppresses mammary tumorigenesis in xenografts in mice by directly interacting with EGFR and inhibiting the EGFR/Ras/Erk/GSK3β signaling pathway. Simultaneously, the downregulation of SHP-1 might be related to the aggressive phenotype of human breast cancers. More importantly, SHP-1 may be used as a new prognostic marker, and targeting the SHP-1-EGFR axis might serve as a promising strategy to enhance therapeutic activity against breast cancer.
