# Supplementary material for: SHP-1 acts as a tumor suppressor by interacting with EGFR and predicts the prognosis of human breast cancer
Source: Cancer Biol Med. 2021 Oct 1;19(4):468–85. doi: 10.20892/j.issn.2095-3941.2020.0501 (PMC9088191; doi:10.20892/j.issn.2095-3941.2020.0501)
Supplement: Supplementary file 1 [file cbm-19-468-s001.pdf]

Supplementary materials

Table S1 Expression of SHP-1 protein in matched paraffin-embedded breast cancer samples and adjacent non-cancerous specimens (*n* = 160)

Pathological type	*n*	SHP-1 expression		*P* value
		High (*n*, %)	Low (*n*, %)	
Breast cancer tissue	160	90 (56.25)	70 (43.75)	<0.0001
Non-cancerous breast tissue	160	119 (74.4)	41 (25.6)	0.012

Table S2 Prognostic factors in the Cox proportional hazard model (*n* = 160)

Parameters	RR	95% CI	Wald	*P* value
Age (years)				
Tumor size (cm)				
Lymph node metastasis	0.903	0.505–1.615	0.118	NS
Histological grade	8.743	2.762–27.676	13.599	<0.001
TNM stage	1.844	0.707–4.811	1.564	NS
ER	0.548	0.244–1.231	2.122	NS
PR	0.684	0.289–1.617	0.748	NS
HER2				
SHP-1 expression	0.512	0.274–0.956	4.413	0.036

$P < 0.05$, indicating statistical significance. RR, relative risk; Wald, Wald value.

Table S3 The relationship between clinicopathological characteristics and SHP-1 expression levels in patients with breast cancer (*n* = 160)

Characteristics		*n*	SHP-1 expression		X^2	*P* value
			High (%)	Low (%)		
Age (years)	≤50	76	41 (53.9)	35 (46.1)	0.312	NS
	>50	84	12 (58.3)	72 (41.7)		
Histological grade	G1	18	6 (33.3)	12 (66.7)	7.812	NS
	G1-G2	26	12 (46.2)	14 (53.8)		
	G2	110	67 (60.9)	43 (39.1)		
	G2-G3	5	4 (80.0)	1 (20.0)		
	G3	1	1 (100)	0 (0.0)		
TNM stage	I	15	12 (80.0)	3 (20.0)	4.213	NS
	II	97	54 (55.7)	43 (44.3)		
	III	48	24 (50.0)	24 (50.0)		
ER	+	102	65 (63.7)	37 (36.3)	6.39	0.013*
	−	58	25 (43.1)	33 (56.9)		
PR	+	79	51 (64.6)	28 (35.4)	4.376	0.04*
	−	81	39 (48.1)	42 (51.9)		
HER-2	+	43	22 (51.2)	21 (48.9)	0.618	NS
	−	117	68 (58.1)	49 (41.9)		
EGFR	High	29	9 (31.0)	20 (69.0)	9.151	0.003**
	Low	131	81 (61.8)	50 (38.2)		

*$P < 0.05$; **$P < 0.01$.

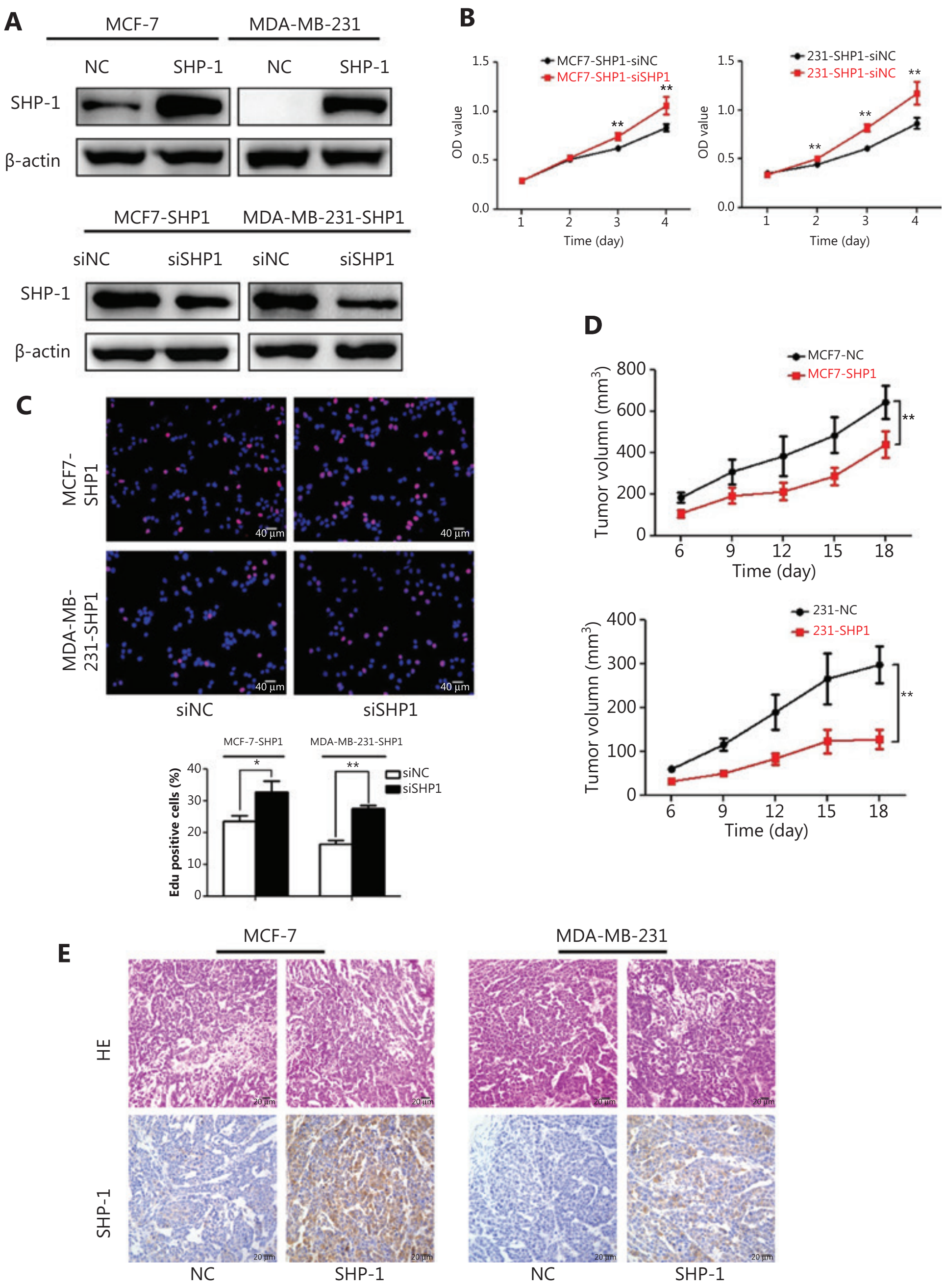

Figure S1 Decreased SHP-1 promoted cell proliferation. (A) Western blot revealed that SHP-1 was efficiently overexpressed by treatment with pLVX-CMV-SHP1 and was knocked down by siSHP1 treatment. (B, C) MTT assays and EdU incorporation assays on MCF-7-SHP1 and MDA-MB-231-SHP1 cells after transfection with siSHP-1 or siNC. Data are presented as mean ± SD for 3 independent experiments. (D) Tumorigenicity assays of cells overexpressing SHP-1. (E) SHP-1 protein was more highly expressed in SHP-1-overexpressing cells than control cells, on the basis of IHC assays. Scale bar, 50 μM (HE), 20 μM (IHC). *P < 0.05; **P < 0.01.

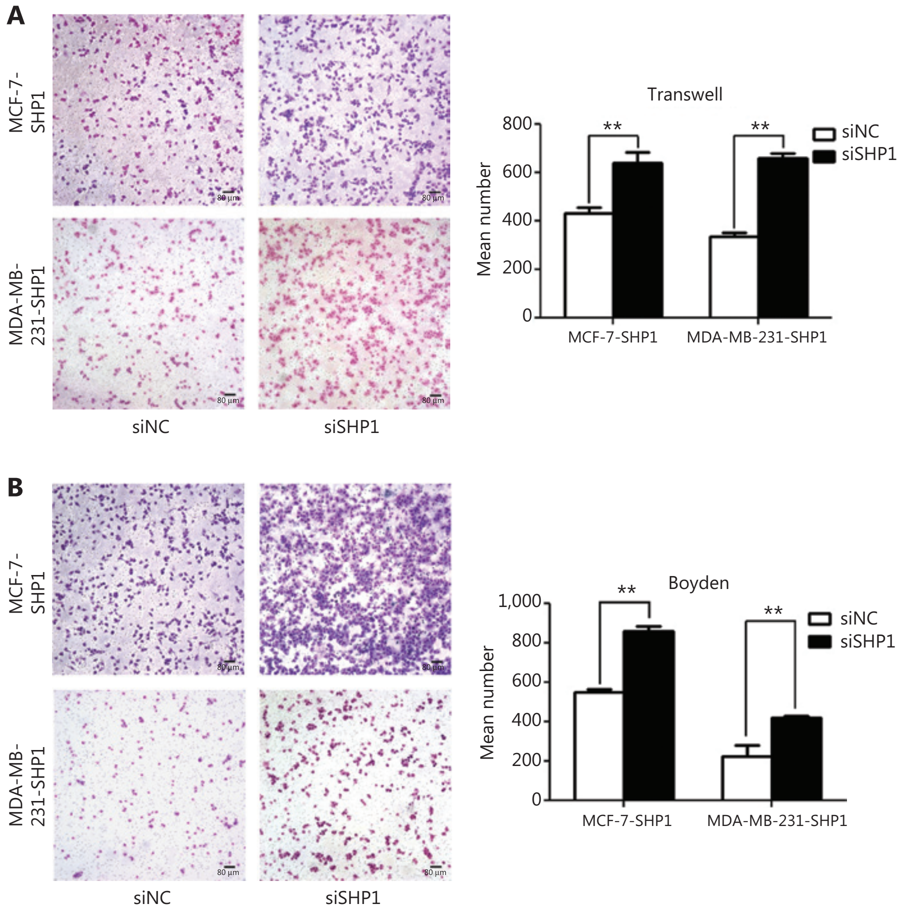

Figure S2 SHP-1 knockdown accelerated cell migration and invasion. (A, B) Downregulation of SHP-1 accelerated cell migration and invasion of MCF-7-SHP1 and MDA-MB-231-SHP1 cells. Data are presented as mean ± SD for 3 independent experiments. **P* < 0.05; ***P* < 0.01 by Student *t* test.

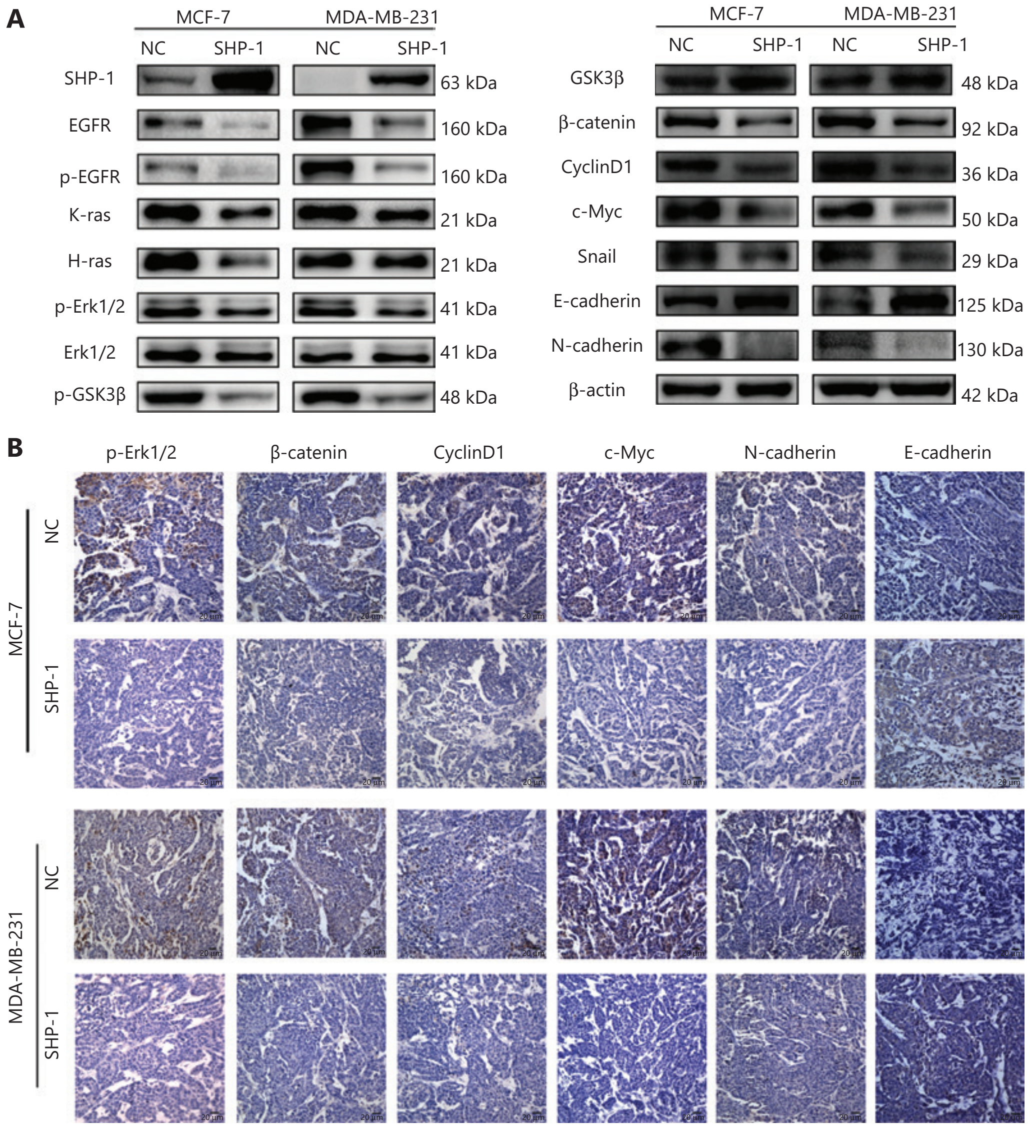

Figure S3 Increased SHP-1 inactivated the Ras/Erk/GSK3β signaling pathway in tumor nodules originating *in vivo*. (A) EGFR, p-EGFR, k-ras, h-ras, phos-Erk1/2, p-GSK3β, β-catenin, cyclin D1, c-Myc, Snail, N-cadherin, GSK3β, and E-cadherin were measured by Western blot in nude mice with subcutaneous overexpression of SHP-1 in tumor tissues. (B) IHC of tumor nodules originating *in vivo* demonstrated that MCF-7-SHP1 and MDA-MB-231-SHP1 cells had lower p-ERK1/2, β-catenin, cyclin D1, c-Myc, and N-cadherin protein levels, and higher E-cadherin protein levels than those in the control groups. All experiments were performed at least in triplicate.
